# Beyond Rebalanced Haemostasis: Haemostatic Phenotypes, Thrombin Generation, and Clinical Risk in Chronic Liver Disease

**DOI:** 10.1055/a-2927-9053

**Published:** 2026-08-24

**Authors:** Sohaib Mukhtar Agouba, Pavla Bradacova, Jan Mikler, Miroslava Drotarova, Kristina Maria Belakova, Monika Brunclikova, Veronika Voskova Gemelova, Jan Stasko, Ingrid Skornova, Tomas Simurda

**Affiliations:** 1 National Centre of Haemostasis and ThrombosisDepartment of Hematology and TransfusiologyComenius University in Bratislava, Jessenius Faculty of Medicine in Martin, University Hospital MartinMartinSlovakia; 2 Department Clinical Hematology 48213 Masaryk Hospital in Usti nad LabemUsti nad LabemCzech Republic; 3 Department of Pediatrics 37864 Comenius University in Bratislava, Jessenius Faculty of Medicine in Martin, University Hospital MartinMartinSlovakia; 4 Department of Clinical Biochemistry 112842 Comenius University in Bratislava Jessenius Faculty of Medicine in Martin, University Hospital MartinMartinŽilina RegionSlovakia

**Keywords:** advanced chronic liver disease, haemostatic phenotypes, thrombin generation, metabolic dysfunction–associated steatotic liver disease, portal vein thrombosis, precision haemostasis

## Abstract

Advanced chronic liver disease (ACLD) profoundly remodels haemostasis through concurrent reductions in procoagulant factors and natural anticoagulants, qualitative platelet abnormalities, elevated von Willebrand factor and factor VIII, and dynamic alterations in fibrinolysis. Although rebalanced haemostasis has transformed understanding of coagulation in cirrhosis, accumulating evidence indicates that haemostatic balance varies according to disease aetiology, fibrosis severity, and clinical stage. This review advances a phenotypic framework for haemostasis in chronic liver disease and explores its implications for risk stratification and antithrombotic management. Three developments support this framework. First, thrombomodulin-modified thrombin generation assays identify distinct haemostatic phenotypes across the disease spectrum, distinguishing the hypercoagulable profile of compensated cirrhosis from hypocoagulable states observed in acute-on-chronic liver failure, whereas metabolic dysfunction–associated steatotic liver disease emerges as a particularly prothrombotic phenotype. Second, recent studies link liver stiffness measurement with thrombin-generation parameters, suggesting convergence between structural liver injury and functional haemostatic assessment. Third, complementary evaluation of fibrin structure, fibrinolysis, plasmin generation, and viscoelastic clot mechanics provides information beyond thrombin kinetics alone, despite important analytical limitations. The review applies this framework to clinically relevant complications, including bleeding, venous thromboembolism, atrial fibrillation, and portal vein thrombosis (PVT). Recent histopathological and clinical evidence challenges the traditional concept of cirrhotic PVT as a manifestation of systemic hypercoagulability and instead supports a predominantly vascular pathogenesis driven by portal-flow abnormalities, endothelial dysfunction, and intimal remodelling. Collectively, these findings support a transition from rebalanced haemostasis towards precision haemostatic phenotyping in ACLD.

## Introduction

Chronic liver disease (CLD) affects an estimated 1.5 billion individuals globally and accounts for approximately 2 million deaths annually (~4% of all deaths), 1 2 3 4 with metabolic dysfunction–associated steatotic liver disease (MASLD) now the leading aetiology in many regions. 5 6 Hepatocellular carcinoma (HCC), the sixth most common malignancy worldwide, typically represents the terminal stage, 7 and MASLD is increasingly recognised as a pivotal and growing risk factor for HCC, particularly across Asia. 8 Beyond hepatic injury, CLD profoundly alters haemostasis in ways that are clinically relevant to thrombosis specialists, transplant teams, and intensivists alike. 9

The conceptual shift from cirrhosis-as-bleeding-disorder to rebalanced haemostasis has been established for over two decades and has been the subject of multiple narrative reviews, position papers, and guidance documents. 10 11 In brief, parallel reductions in procoagulant factors and natural anticoagulants, qualitative platelet dysfunction offset by elevated von Willebrand factor (vWF), and stage-dependent fibrinolytic changes produce a fragile equilibrium that conventional tests—assessing only the initiation phase of clotting in platelet-poor plasma—cannot resolve. 12 13 14 Thrombomodulin (TM)-modified thrombin generation assays (TGA), viscoelastic testing, and fibrin- and plasmin-based methods have collectively reshaped the conceptual landscape, demonstrating that thrombin-generating capacity is often preserved or increased despite prolonged prothrombin time (PT)/international normalised ratio (INR). 15

The clinical consequences of this paradigm shift remain incompletely worked out; however, and three open questions motivate the present review. First, haemostatic phenotype in CLD is increasingly recognised as heterogeneous: compensated cirrhosis, decompensated disease, and acute-on-chronic liver failure (ACLF) display systematically different TGA signatures, 15 16 17 and aetiology matters—MASLD in particular exhibits a distinct prothrombotic profile driven by elevated factor VIII, fibrinogen, and PAI-1, 18 19 with cardiovascular implications that extend beyond classical cirrhotic thrombotic events. Second, fibrosis severity itself, as measured noninvasively by liver stiffness, has recently been shown to correlate with TGA parameters in compensated disease, 15 raising the possibility that imaging and global haemostatic assays converge on a shared risk-stratification axis. Third, the framing of portal vein thrombosis (PVT)—historically attributed to systemic hypercoagulability—is undergoing revision, with growing histological and clinical evidence that cirrhotic PVT is predominantly a vascular disease, characterised by intimal hyperplasia and altered portal flow rather than classical thrombus formation. 20 21

These shifts demand a phenotypic rather than uniform approach to coagulation assessment, transfusion, and antithrombotic management in CLD. The aims of this review are therefore 3-fold. First, to consolidate the mechanistic basis of rebalanced haemostasis with explicit attention to its fragility across etiologies and stages, including the metabolic prothrombotic phenotype that distinguishes MASLD. Second, to critically appraise the global assays—TGA (including standardised platforms such as ST Genesia and emerging whole-blood approaches), fibrin-structure, and fibrinolysis assays including the plasmin generation assay (PGA), and viscoelastic methods—with their respective strengths, limitations, and analytic blind spots. Third, to translate this phenotypic framework into specific clinical decisions around bleeding, PVT, atrial fibrillation (AF), and antithrombotic therapy, with particular focus on MASLD-associated disease, on the reconceptualisation of cirrhotic PVT, and on emerging biomarkers (liver stiffness, hepatokines) that may bridge structural and functional risk assessment.

This narrative review was based on a literature search of PubMed and Web of Science from January 2010 through April 2026, using combinations of the following terms: chronic liver disease, cirrhosis, MASLD, haemostasis, rebalanced haemostasis, thrombin generation, platelet, fibrinolysis, thrombosis, PVT, viscoelastic testing, and anticoagulation. Reference lists of identified articles and current clinical practice guidelines (European Association for the Study of the Liver [EASL], American Association for the Study of Liver Diseases, International Society on Thrombosis and Haemostasis Scientific and Standardization Committee [ISTH SSC], Baveno VII) were hand-searched. The forthcoming Baveno VIII consensus was tracked through prepublication sources. Priority was given to randomised trials, systematic reviews, and large prospective cohorts published in the past 5 years. Older landmark studies were retained where they remain authoritative. The search was limited to English-language publications.

## Chronic Liver Disease: A Brief Primer

CLD is a dynamic continuum of progressive hepatic injury in which sustained insults—including steatosis—drive the transition from fibrosis to advanced chronic liver disease (ACLD), culminating in compensated and decompensated disease and ultimately HCC. The ACLD framework reconceptualises cirrhosis as a late histological endpoint and instead describes ACLD as a haemodynamic and biological continuum of progressive fibrosis, portal hypertension, and functional impairment. Clinical decision-making in ACLD has progressively moved from histologically defined cirrhosis towards the ACLD framework, divided into compensated ACLD and decompensated ACLD stages. Ongoing consensus discussions have highlighted a possible shift towards reserving ‘cirrhosis’ for histological diagnosis; at present, this is a proposed trend, not an enacted guideline. The 2023 nomenclature revision replaced nonalcoholic fatty liver disease with MASLD, NASH with metabolic dysfunction–associated steatohepatitis (MASH), and introduced metabolic dysfunction– and alcohol-related liver disease for mixed metabolic–alcohol etiologies. 5 6 Within this framework, clinically significant portal hypertension (CSPH) is the central prognostic determinant, often outperforming histology, 22 23 and is highly relevant to haemostatic phenotyping because portal hypertension, endothelial dysfunction, and systemic inflammation variably influence bleeding and thrombotic risk. 13 24 25 Noninvasive assessment is now preferred over biopsy for fibrosis staging 26 : Liver stiffness measurement (LSM) by transient elastography is the principal surrogate for fibrosis severity: values <8 kPa effectively exclude, and ≥12 to 15 kPa strongly suggest, advanced fibrosis. 9 27 28 The Baveno VII ‘Rule of Five’ (liver stiffness thresholds of 10, 15, 20, and 25 kPa) is used to stratify the risk of CSPH and clinical decompensation. The ANTICIPATE and ANTICIPATE-NASH models—which combine LSM with platelet count, and additionally body mass index in MASLD—provide individualised quantitative estimates of CSPH risk. In MASLD with obesity, 29 transient elastography–based stratification is less reliable, and the ANTICIPATE-NASH model incorporating body mass index is preferred. 22 29 30 These models show good discriminatory performance (area under the receiver operating characteristic curve ~0.80 to 0.91 across derivation/validation cohorts), although external validation remains more limited for ANTICIPATE-NASH than for the original ANTICIPATE model. Beyond fibrosis staging and portal hypertension risk stratification, higher LSM values are also associated with subsequent liver-related events (typically including hepatic decompensation, HCC, liver transplantation, or liver-related death), supporting their role as clinically meaningful risk markers rather than purely anatomical fibrosis surrogates. 29 Spleen stiffness measurement (SSM), preferably using a dedicated 100 Hz probe, further refines CSPH assessment. 22 31 Serum-based tests including the fibrosis-4 index (FIB-4), enhanced liver fibrosis score, FibroTest, and FibroMeter provide alternative risk stratification. 26 This stratification is now therapeutically actionable: in patients with CSPH, nonselective β-blockers—preferentially carvedilol—are recommended to reduce the risk of first hepatic decompensation (PREDESCI trial; Baveno VII). 22 32 For thrombosis specialists, these metrics matter because LSM and SSM not only stage fibrosis and portal hypertension but also correlate with the prothrombotic shift seen on thrombin generation testing, suggesting that noninvasive imaging and global haemostatic assays may converge on a shared risk-stratification framework. 15 33 34 ACLD severity for prognosis and transplant allocation is assessed using the Child–Pugh classification 35 and the model for end-stage liver disease (MELD) and its variants—MELD-Na, MELD 3.0, and equity-adjusted models such as the Gender-Equity Model for Allocation—which estimate short-term mortality and guide organ prioritisation. 35 36 In alcohol-related disease, Maddrey’s discriminant function, the Lille score, and the age–bilirubin–INR–creatinine (ABIC) score guide management decisions. 37 Current EASL guidance supports a multimodal approach that integrates noninvasive tests, validated scoring systems, and clinical context for optimal staging and prognostication. 25 38 The principal severity and staging tools used in CLD, with their components and haemostatic relevance, are summarised in Table 1 .

**Table 1:** Severity and staging tools in chronic liver disease and their haemostatic relevance.

Score/tool	Components	Primary clinical use
Child–Pugh 35	Bilirubin, albumin, INR, ascites, encephalopathy	Cirrhosis severity classification (A/B/C); prognosis; eligibility for interventions including anticoagulation
MELD 35	Bilirubin, creatinine, INR	Liver transplant allocation; prediction of 3-mo mortality
MELD-Na 35	MELD + serum sodium	Transplant allocation with hyponatraemia adjustment
MELD 3.0 35	Bilirubin, creatinine, INR, sodium, albumin, sex	Updated (2022) transplant allocation model
GEMA/GEMA-Na 36	Gender-equity adjusted MELD variants	Transplant allocation with sex-equity correction
CLIF-C ACLF 39	Six organ systems, including coagulation (INR ≥ 2.5)	Mortality prediction in acute-on-chronic liver failure
CLIF-C AD 39	Age, sodium, white cell count, creatinine, INR	Mortality prediction in acute decompensation (without ACLF)
BCLC 40 41	Tumour burden, liver function (Child–Pugh), performance status	HCC staging and treatment allocation
Baveno VII criteria 22	Liver stiffness (TE) + platelet count	Diagnosis of clinically significant portal hypertension; variceal screening
Transient elastography (TE/FibroScan) 9 27 28	Liver stiffness (kPa)	Noninvasive fibrosis staging; CSPH assessment
FIB-4 26	Age, AST, ALT, platelet count	First-line noninvasive fibrosis risk stratification, endorsed by 2024 EASL–EASD–EASO guidance
ELF score 26	TIMP-1, PIIINP, hyaluronic acid	Serum-based fibrosis staging
FibroTest/FibroMeter 26	Multimarker serum panels	Serum-based fibrosis staging
Maddrey’s discriminant function 37	Bilirubin, prothrombin time	Severe alcohol-associated hepatitis—threshold for corticosteroid therapy
Lille score 37	Age, albumin, bilirubin, creatinine, PT, change in bilirubin at day 7	Assessment of steroid response in alcohol-associated hepatitis
ABIC 37	Age, bilirubin, INR, creatinine	Survival prediction in alcohol-associated hepatitis

Abbreviations: ACLF, acute-on-chronic liver failure; AD, acute decompensation; ABIC, age–bilirubin–INR–creatinine; AST, aspartate aminotransferase; ALT, alanine aminotransferase; BCLC, Barcelona Clinic Liver Cancer; CSPH, clinically significant portal hypertension; DOAC, direct oral anticoagulant; EASD, European Association for the Study of Diabetes; EASL, European Association for the Study of the Liver; EASO, European Association for the Study of Obesity; ELF, enhanced liver fibrosis; ETP, endogenous thrombin potential; FIB-4, fibrosis-4 index; GEMA, gender-equity model for allocation; HCC, hepatocellular carcinoma; INR, international normalised ratio; MELD, model for end-stage liver disease; PIIINP, procollagen III amino-terminal propeptide; PT, prothrombin time; PVT, portal vein thrombosis; TE, transient elastography; TIMP-1, tissue inhibitor of metalloproteinase-1.

The INR is incorporated into several severity scores (Child–Pugh, MELD and derivatives, CLIF-C, ABIC, Maddrey, Lille), where it reflects impaired hepatic synthesis of procoagulant factors. However, INR does not capture the concomitant reduction in natural anticoagulants or the contributions of platelets, vWF, and fibrinolytic pathways, and therefore does not reflect overall haemostatic balance. 33 42 43

## Pathophysiology of Haemostatic Rebalance in Chronic Liver Disease

### Overview

**Fig. 1:**
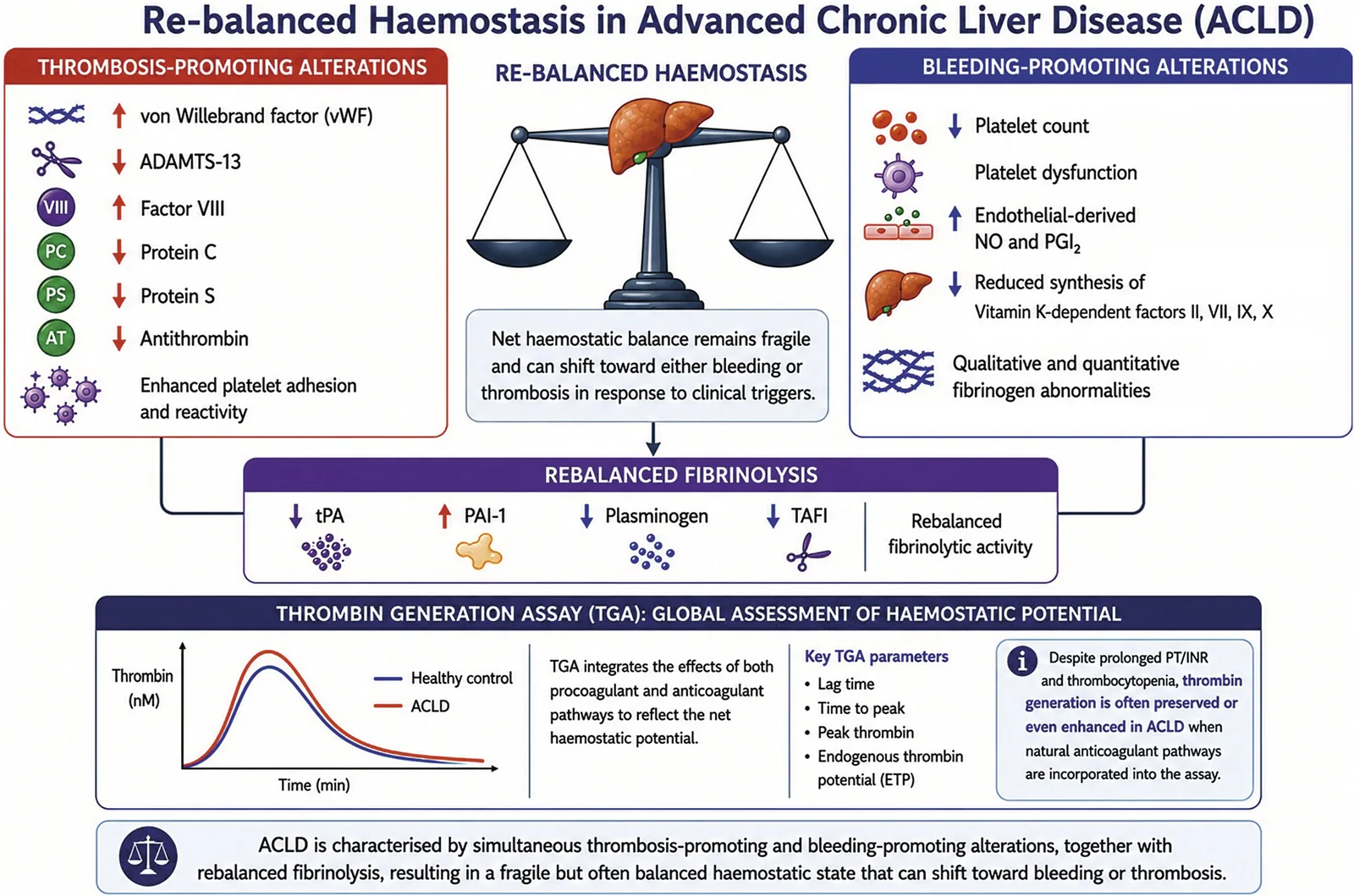
Conceptual synthesis building on Lisman and Porte 2010 and subsequent ISTH SSC communications. 13 ACLD is characterised by concurrent changes in procoagulant, anticoagulant, and fibrinolytic pathways. Reduced hepatic synthesis of coagulation factors (FII, FV, FVII, FIX, FX, FXI) is counterbalanced by increased von Willebrand factor (vWF) and reduced ADAMTS-13 activity. 42 47 48 Prothrombin time (PT) and activated partial thromboplastin time (aPTT) are characteristically prolonged but reflect only the procoagulant limb of haemostasis and do not capture the rebalanced state. Fibrinolysis is modulated by tissue plasminogen activator (tPA), α2-antiplasmin, and thrombin-activatable fibrinolysis inhibitor (TAFI). Anticoagulant pathways involve reduced antithrombin (AT) and altered protein C pathway (PC/PS), with additional effects of nitric oxide (NO). Global coagulation is reflected by thrombin generation parameters, including endogenous thrombin potential (ETP), lag time, time to peak, and peak thrombin. 13 49 Fibrinogen abnormalities in CLD include both quantitative hypofibrinogenaemia, reflecting reduced fibrinogen concentration in advanced disease, and qualitative acquired dysfibrinogenaemia, reflecting impaired fibrinogen function and altered fibrin polymerisation despite preserved or even increased antigenic fibrinogen levels. 50 51 ACLD, advanced chronic liver disease; ISTH SSC, International Society on Thrombosis and Haemostasis Scientific and Standardization Committee.

Patients with ACLD are at risk of both bleeding and thrombosis, including venous thromboembolism (VTE), PVT, ischaemic stroke, and cardiovascular events. 44 45 This dual risk reflects rebalanced haemostasis—a fragile equilibrium of concurrent reductions in procoagulant and anticoagulant factors, qualitative platelet dysfunction, elevated vWF, preserved or increased factor VIII activity, 44 and parallel changes in fibrinolytic regulators, 11 12 13 46 producing a state of fragile equilibrium that is best understood as rebalanced rather than absent haemostasis ( Fig. 1 ).

### Primary Haemostasis

Primary haemostasis in CLD is characterised by quantitative thrombocytopenia, qualitative platelet dysfunction, and compensatory changes in vWF biology. 13 42 49 52 Thrombocytopenia is multifactorial, reflecting splenic sequestration secondary to portal hypertension, reduced hepatic production of thrombopoietin, immune-mediated platelet destruction, and bone marrow suppression related to alcohol, viral infection, systemic inflammation, or medications. 53 54 55 Platelet function may be impaired through abnormalities in adhesion, aggregation, secretion, and signaling, particularly in advanced or decompensated disease. 53 54 56 However, platelet activity is not uniformly reduced, and an active debate has emerged regarding the dominant platelet phenotype in CLD. Thrombin generation and whole-blood aggregometry studies indicate that, after adjustment for platelet count, the platelet contribution to haemostasis may be preserved or even enhanced, 57 58 and several recent studies have argued more strongly that platelet hyperreactivity rather than hypofunction dominates outside the setting of acute decompensation—supported by elevated circulating markers of platelet activation (soluble CD40L, P-selectin, urinary 11-dehydro-thromboxane B2) and enhanced agonist responsiveness, particularly in MASLD and stable compensated cirrhosis. 59 60 61 62 63 This reframing has implications both for thrombotic risk and for fibrogenesis, in which activated platelets are increasingly implicated as drivers of hepatic stellate-cell activation and extracellular matrix deposition. 64 65 66 Consistent with this, platelet hyperreactivity in MASLD has been shown to correlate with hepatic fat content, iron deposition, and liver volume, and preliminary data suggest that antiplatelet therapy may reduce hepatic volume and iron accumulation in this setting. 63 Despite these abnormalities, primary haemostasis is often partially preserved through compensatory increases in circulating vWF, 13 released from endothelial Weibel–Palade bodies in response to endothelial activation, inflammation, and portal hypertension. 52 Elevated vWF enhances platelet adhesion under high-shear conditions and may partially offset thrombocytopenia. 13 42 In parallel, ADAMTS-13, a vWF-cleaving protease produced in part by hepatic stellate cells, is reduced in cirrhosis, and the resulting persistence of ultralarge vWF multimers further enhances platelet recruitment despite low platelet counts. 42 47 48 The functional consequences of these alterations can be explored using the Platelet Function Analyzer (PFA-200), a whole-blood assay that measures platelet-dependent closure time under high-shear conditions. Because the assay integrates platelet count, haematocrit, platelet reactivity, and circulating vWF levels, it may in principle provide an integrated readout of the primary haemostatic axis in cirrhosis. In practice, however, the elevated vWF concentrations characteristic of CLD partially normalise closure times despite substantial alterations in platelet biology, and current evidence does not support PFA-200 for reliable discrimination between bleeding-prone and thrombotic phenotypes or for routine haemostatic risk stratification in CLD. 67 Primary haemostasis in CLD therefore reflects a rebalanced interaction between reduced platelet mass and enhanced vWF-mediated recruitment. 13 This explains why platelet count alone poorly predicts spontaneous bleeding in stable cirrhosis. 58 68 However, this compensation is fragile and may fail during bacterial infection, 69 70 acute kidney injury, 71 or acute decompensation and ACLF, 72 contributing to bleeding susceptibility in these contexts.

### Coagulation Factors and Natural Anticoagulants

**Fig. 2:**
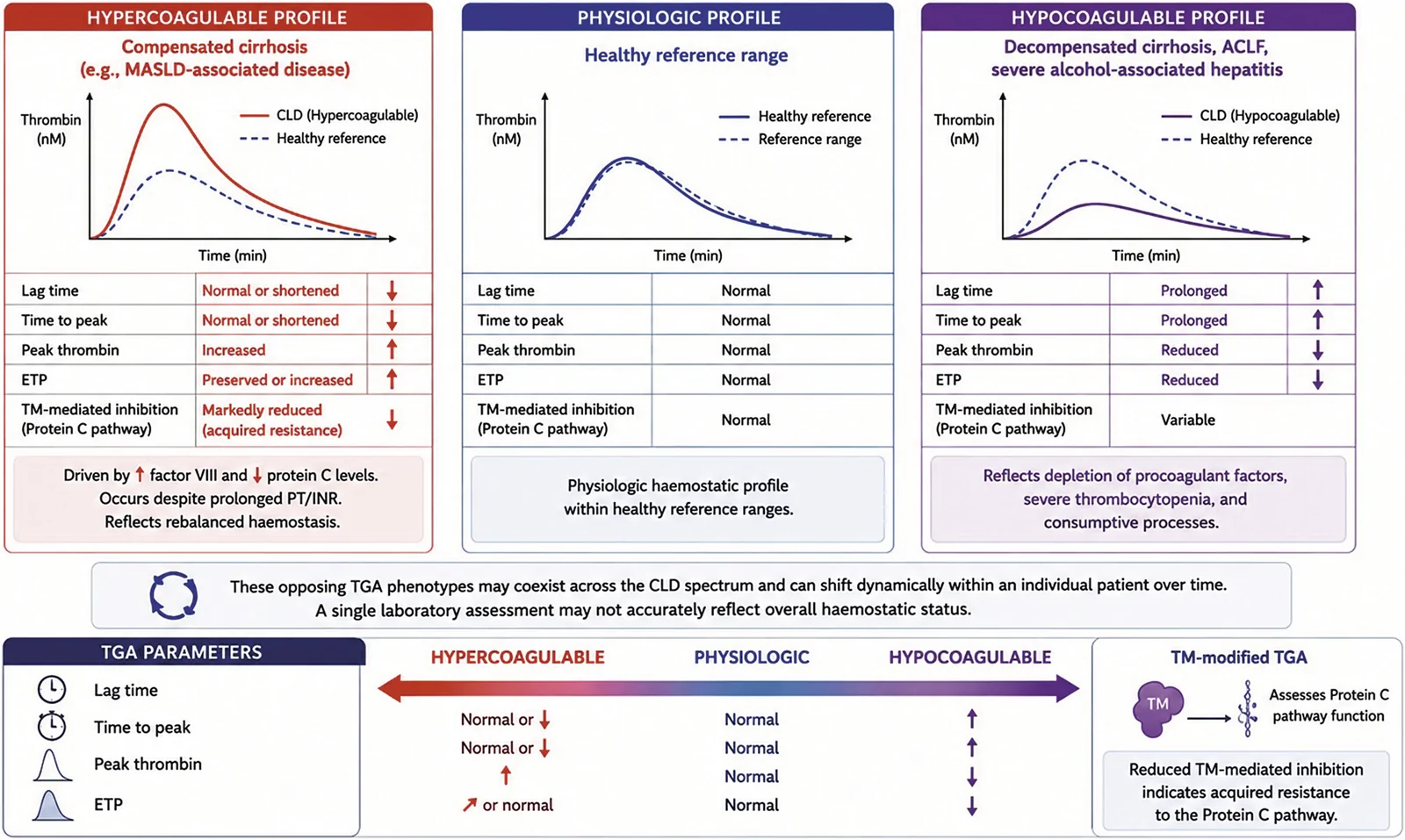
Thrombin generation assay profiles across the spectrum of chronic liver disease. Three etiology- and stage-specific haemostatic phenotypes are illustrated: a hypercoagulable shift typical of compensated cirrhosis and MASLD-associated disease (elevated peak thrombin and ETP, preserved or shortened lag time); a physiologic profile within healthy reference ranges; and a hypocoagulable phenotype characteristic of acute-on-chronic liver failure and severe alcohol-associated hepatitis (reduced peak thrombin and ETP, prolonged lag time). ACLF, acute-on-chronic liver failure; CLD, chronic liver disease; ETP, endogenous thrombin potential; PT/INR, prothrombin time/international normalised ratio; TGA, thrombin generation assay; TM, thrombomodulin.

Most coagulation factors are synthesised hepatically and decline progressively with worsening liver function, including the vitamin K–dependent factors II, VII, IX, and X, factor V, and contact-pathway factors XI and XII. 73 Factor VII, with its short half-life, declines early and contributes substantially to PT/INR prolongation. 74 This procoagulant decline is counterbalanced by parallel decreases in natural anticoagulants—particularly protein C, protein S, and antithrombin—also hepatically synthesised. 14 The net haemostatic state therefore cannot be inferred from PT/INR or activated partial thromboplastin time (aPTT). 33 A major exception is factor VIII, which is typically preserved or elevated. Factor VIII is largely endothelial-derived and rises with endothelial activation, inflammation, and reduced clearance. 75 Together with elevated vWF and reduced protein C, increased factor VIII contributes to preserved or enhanced thrombin generation. 76 This concept is supported by thrombomodulin-modified TGA, which incorporates the protein C pathway and demonstrates preserved or increased thrombin generation despite prolonged PT/INR—the central biochemical basis of rebalanced haemostasis ( 14 76 ; Fig. 2 ).

### Fibrinolytic System

Fibrinolysis in CLD is altered across multiple components, and the net phenotype is variable and stage-dependent. 72 Plasminogen, the zymogen precursor of plasmin, is reduced in advanced disease—a change that, considered in isolation, would tend to limit fibrinolytic capacity rather than promote it. However, the principal physiological inhibitors of fibrinolysis—α _2_ -antiplasmin and thrombin-activatable fibrinolysis inhibitor (TAFI)—are also reduced, lowering the threshold for plasmin activity once it is generated. 72 Circulating tissue plasminogen activator (tPA) is often increased, reflecting both endothelial activation and reduced hepatic clearance. 77 PAI-1 levels are more variable: elevated in inflammatory and metabolic phenotypes including MASLD/MASH, where it contributes directly to the prothrombotic signature of metabolic liver disease, 18 19 73 78 but disproportionately low relative to tPA in advanced decompensated cirrhosis. 11 21

The net fibrinolytic phenotype therefore depends on the balance of these opposing changes rather than on any single parameter. In most patients with stable compensated disease the fibrinolytic system remains in a fragile rebalanced state. A subset—particularly those with decompensated cirrhosis, ACLF, infection, renal dysfunction, or severe systemic inflammation—develop clinically relevant hyperfibrinolysis, driven by markedly reduced α _2_ -antiplasmin, and TAFI together with elevated tPA, despite the concurrent reduction in plasminogen. 16 79 Functionally, this is reflected in shortened clot lysis time (CLT) and increased PG and helps explain why bleeding susceptibility may emerge in advanced disease even when thrombin generation appears preserved. 16 The PGA, a functional test analogous in concept to TGA but reporting plasmin kinetics, can identify hyperfibrinolysis when conventional and viscoelastic measurements are equivocal and provides a mechanistic readout that integrates plasminogen activation, tPA/PAI-1 balance, and antiplasmin reserve 80 81 ; its application in CLD is discussed in Section Fibrin Structure and Fibrinolysis Assays.

### Endothelial Dysfunction

Endothelial dysfunction is a central component of haemostatic imbalance in CLD. Liver sinusoidal endothelial cells (LSECs) regulate vascular tone, sinusoidal resistance, inflammation, and coagulation–fibrinolysis crosstalk. LSEC capillarisation and dysfunction, hepatic stellate cell activation with sinusoidal contraction, impaired nitric oxide signaling, oxidative stress, and inflammatory activation contribute to increased intrahepatic vascular resistance and portal hypertension. Systemic endothelial activation is reflected by increased vWF, factor VIII, soluble adhesion molecules, and altered nitric oxide bioavailability—changes that amplify platelet adhesion, promote thrombin generation, and link local hepatic vascular dysfunction to systemic thromboinflammatory risk. 82

Plasma levels of vWF:Ag and vWFpp correlate well with Child–Pugh classification in chronic hepatitis B, supporting their utility in diagnosing decompensated liver cirrhosis. 83 Behairy et al. further validated plasma vWF:Ag as a reliable, noninvasive marker for grading liver fibrosis in pediatric CLD. 84

Beyond the sinusoidal compartment, emerging evidence implicates portal vein endothelial dysfunction in PVT pathogenesis (revisited in Section Portal Vein Thrombosis) through altered anticoagulant surface properties, inflammatory activation, tissue factor expression, and impaired local fibrinolytic regulation. 72 77

**Fig. 3:**
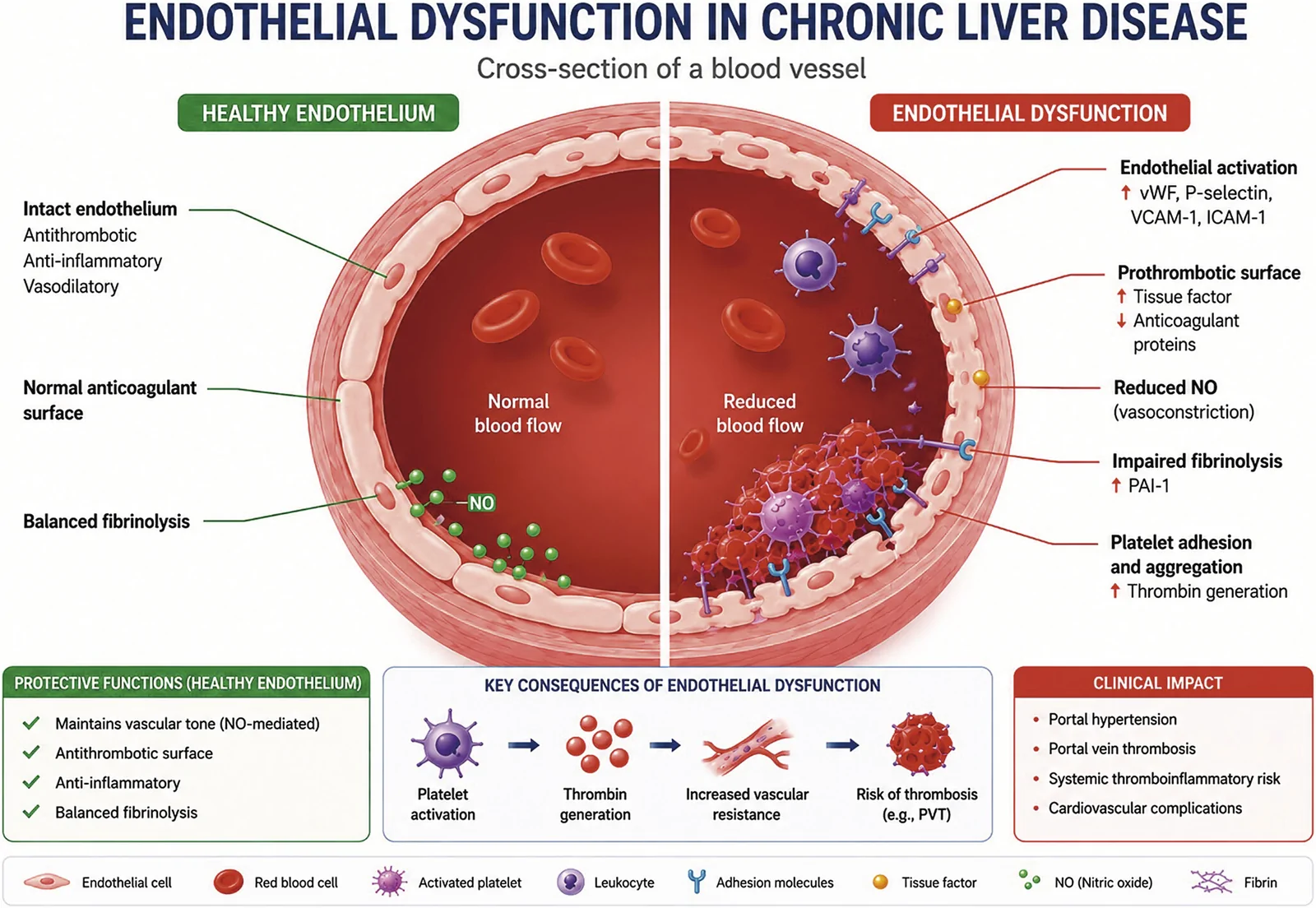
Endothelial dysfunction as a driver of thromboinflammation and vascular complications in chronic liver disease. ICAM-1, intercellular adhesion molecule-1; NO, nitric oxide; PAI-1, plasminogen activator inhibitor-1; PVT, portal vein thrombosis; VCAM-1, vascular cell adhesion molecule-1; vWF, von Willebrand factor.

Endothelial dysfunction may also contribute to extrahepatic vascular disease, including atherosclerosis and cardiovascular risk in metabolic phenotypes, representing a unifying mechanism linking portal hypertension, altered haemostasis, PVT, and systemic vascular complications. 72 85 The endothelial compartment in CLD therefore acts as an active driver of thromboinflammation rather than a passive surface, integrating inflammatory activation, anticoagulant-surface loss, and impaired fibrinolytic regulation with portal-hypertensive haemodynamic stress ( Fig. 3 ).

### Integrated Framework

These mechanistic strands converge into the integrated rebalanced-haemostasis framework illustrated in Fig. 1 , which summarises the concurrent procoagulant, anticoagulant, and fibrinolytic alterations and their relationship to TGA parameters.

## Assessment of Haemostasis in Chronic Liver Disease

### Historical Context

Modern understanding of coagulation evolved from the early 20th-century Morawitz model of tissue factor–mediated thrombin generation, through the cascade model of Macfarlane and of Davie and Ratnoff (1964), to the introduction of continuous fluorogenic TGAs by Hemker and colleagues in the 1990s, culminating in the calibrated automated thrombogram (clotting time [CT]). 86 87 88 89 The clinical relevance of TGA became particularly evident in disorders where conventional tests are misleading—most notably CLD—where thrombomodulin-modified TGA demonstrated preserved or increased thrombin generation despite abnormal PT/INR. 18

### Limitations of Conventional Coagulation Tests

PT (expressed as INR) and aPTT assess only a limited fraction of procoagulant activity in platelet-poor plasma. 90 They do not capture the contributions of platelets, endothelial interactions, natural anticoagulants (protein C, protein S, antithrombin), or the fibrinolytic system, 14 and reflect only the initiation phase of clot formation—approximately 5% of total thrombin generation—therefore correlating poorly with in vivo haemostasis in ACLD. 42 91 This underlies the central paradox of cirrhotic coagulopathy: CTs are prolonged despite preserved or even increased thrombin generation. Despite this, INR-guided preprocedural correction remains common, although increasingly recognised as ineffective and potentially harmful—prophylactic plasma transfusion does not reliably normalise INR and may exacerbate volume overload and increase portal pressure. 92 93 94 95 Current ISTH SSC guidance explicitly cautions against using conventional tests to guide anticoagulation or transfusion decisions in cirrhosis. 96 97

### Thrombin Generation Assays

Global functional assays—particularly TGA with thrombomodulin modification—provide an integrated assessment of haemostatic balance. 14 The thrombin generation curve describes the dynamic phases of coagulation (initiation, amplification, termination) and yields kinetic and quantitative parameters reflecting the integrated action of procoagulant factors, natural anticoagulants engaged by added thrombomodulin, and plasma inhibitors. 90

#### Hypo/Hypercoagulable Profiles in Chronic Liver Disease

TM-modified TGA reveals two contrasting haemostatic phenotypes in CLD, as illustrated in Fig. 2 . In stable compensated cirrhosis, endogenous thrombin potential (ETP) and peak thrombin are preserved or increased, often accompanied by accelerated thrombin generation kinetics, and TM-mediated inhibition is markedly reduced—reflecting an acquired resistance to the protein C pathway driven by elevated factor VIII and reduced protein C levels. 13 42 76 This hypercoagulable profile exists despite prolonged PT/INR and underpins the concept of rebalanced haemostasis. 13 42 In contrast, decompensated cirrhosis, ACLF, and sepsis are more frequently associated with a hypocoagulable TGA profile, characterised by reduced peak thrombin and ETP, and prolonged lag time. 16 17 77 These changes reflect cumulative depletion of procoagulant factors, severe thrombocytopenia, and consumptive processes. 16 These opposing TGA phenotypes may coexist across the CLD spectrum and can shift dynamically within an individual patient over time, explaining why a single laboratory assessment may not accurately reflect overall haemostatic status. 12 72 79 82 These parameters and their typical interpretation across CLD stages are summarised in Table 2 .

**Table 2:** Thrombin generation assay parameters and interpretation in chronic liver disease.

Parameter	Definition	Interpretation in chronic liver disease
Lag time (min)	Delay before thrombin generation begins	Prolonged in decompensated cirrhosis or severe factor deficiency; also prolonged by anticoagulant exposure such as VKA/DOACs. Shortened with accelerated thrombin initiation 98 99
Time to peak (min)	Time to maximal thrombin concentration	required to reach maximal thrombin generation; prolonged in decompensated/Child–Pugh C cirrhosis in whole-blood TGA, but may be shortened in plasma-based assays showing hypercoagulable profiles 100
Peak thrombin (nM)	Maximum thrombin concentration achieved	Maximal thrombin concentration reached; may be preserved or increased in stable/nonbleeding cirrhosis, but reduced in decompensated cirrhosis, 101 severe thrombocytopenia/anaemia, and some ACLF or septic inflammatory phenotypes 14 100 102
Endogenous thrombin potential (ETP, nM·min)	Area under the thrombin generation curve	Area under the thrombin-generation curve; most informative when measured with thrombomodulin to engage the protein C pathway. Typically preserved or increased in stable cirrhosis, reflecting reduced anticoagulant reserve/protein C pathway impairment 101 103
Velocity index	Rate of thrombin burst (slope of propagation phase)	Rate of thrombin burst propagation; may be increased in compensated cirrhosis or TM-modified hypercoagulable profiles, but reduced in decompensated or hypocoagulable whole-blood TGA phenotypes 100 101

Abbreviations: ACLF, acute-on-chronic liver failure; DOAC, direct oral anticoagulant; TGA, thrombin generation assay; TM, thrombomodulin; VKA, vitamin K antagonist.

Note: All interpretations assume standardised assay conditions; ETP, in particular, is the most physiologically informative when measured with thrombomodulin to engage the protein C pathway. 18 49 104

#### Assay-Dependence and Interlaboratory Variability

TGA performance is highly assay-dependent, and this remains the principal practical barrier to broader clinical adoption. Tissue factor concentration, phospholipid composition, calcium concentration, and the source and dose of recombinant thrombomodulin all influence absolute thrombogram parameters and the magnitude of protein C-pathway engagement. 49 With the historically dominant calibrated automated thrombogram (CT) method, interlaboratory comparability of ETP, peak thrombin, and TM-modified ratios is poor in the absence of locally agreed protocols, 105 which has limited the pooling of single-centre CLD studies and constrained the development of generalisable cut-offs.

A separate conceptual issue is that the clinical meaning of TGA values in CLD cannot be inferred from comparison with healthy-donor reference ranges. Patients with cirrhosis sit at a different haemostatic equilibrium—concurrent reductions in procoagulant and anticoagulant capacity, altered protein C–pathway responsiveness, and elevated factor VIII—so values that depart from a healthy reference interval may carry no equivalent thrombotic or bleeding implication. The clinically relevant comparisons in CLD are therefore between disease stages, etiologies, and within-patient trajectories rather than against healthy reference intervals. 12 13 18 82 106

The ST Genesia automated platform addresses much of the CT-related standardisation problem through fixed reagent composition, integrated calibration against a normal plasma reference, and automated correction for inner-filter and substrate-consumption effects. 107 Studies in CLD using ST Genesia report improved interrun and intersite reproducibility and have begun to define disease-specific thrombogram patterns, including the stiffness-correlated profile in compensated cirrhosis. 15 Platelet-rich plasma–based TGA partially overcomes the limitations of platelet-poor plasma assays by incorporating the contribution of circulating platelets to thrombin generation. However, variability related to platelet count, platelet activation status, and preanalytical processing has limited standardisation and currently prevents widespread clinical implementation in CLD. Whole-blood TGA platforms, which preserve cellular contributions absent from platelet-poor plasma assays, are an additional area of methodological progress; recent comparative data caution, however, that plasma and whole-blood TGA capture overlapping but nonidentical aspects of haemostatic capacity in CLD, 108 so the two modalities are best regarded as complementary rather than interchangeable.

#### Methodological and Clinical Validation Limitations

Beyond interlaboratory variability, several methodological constraints limit the clinical use of TGA in CLD. Standard assays are performed in platelet-poor plasma, missing the cellular and endothelial contributions that shape in vivo haemostasis 49 ; whole-blood TGA platforms are emerging but not widely adopted. 109 Preanalytical variables—anticoagulant choice, time to processing, freezing, and thawing—can alter results substantially, particularly in patients with thrombocytopenia or elevated FVIII. 49 Reagent costs, technical complexity, and the need for trained personnel further restrict TGA to specialised laboratories. Most importantly, although TGA has provided crucial mechanistic insight into rebalanced haemostasis, no prospective trial has validated TGA-guided transfusion, prophylaxis, or anticoagulation in CLD. 82 TGA therefore remains a research and phenotyping tool rather than a clinical decision-support assay, and its findings should be interpreted alongside clinical context, viscoelastic testing, and conventional parameters rather than in isolation. 49 82

#### Aetiology- and Stage-Specific Thrombin Generation Assay Phenotypes in Chronic Liver Disease

Beyond the broad compensated-versus-decompensated distinction, emerging evidence suggests that TGA findings in CLD vary by aetiology and clinical context, with implications for risk stratification. In MASLD, particularly with metabolic comorbidity and advanced fibrosis, TGA appears to show a more pronounced procoagulant shift than viral- or alcohol-related disease—driven by elevated factor VIII, fibrinogen, and PAI-1, alongside relatively preserved or only modestly reduced protein C 19 110 —although direct stage-matched comparisons across etiologies remain limited. This pattern aligns with the increased cardiovascular and thrombotic risk observed clinically in MASLD and supports the argument that aetiology-specific phenotyping may refine global thrombotic risk assessment in CLD. In alcohol-related liver disease, TGA findings track decompensation status more closely than histological stage; severe alcohol-associated hepatitis exhibits a hypocoagulable profile with reduced peak thrombin and ETP, whereas abstinence and clinical recovery partially restore thrombin-generating capacity. Viral etiologies (hepatitis C virus [HCV], hepatitis B virus) historically contributed most of the foundational TGA literature in CLD and most clearly exemplify the classical rebalanced phenotype; the impact of direct-acting antivirals on long-term TGA recovery remains incompletely characterised.

In acute decompensation and ACLF ( Fig. 2 ), the interpretation of TGA requires careful attention to the clinical state and timing at which it is performed. Hospitalisation, infection, and organ failure precipitate acute hypocoagulable shifts driven by procoagulant factor consumption, severe thrombocytopenia, and inflammatory dysregulation, with partial recovery during clinical resolution. 16 Comparative data position ACLF haemostasis as partially overlapping with that of sepsis: Lisman et al. found concordance of haemostatic alterations between the two conditions, with evidence of preserved haemostatic capacity in both groups, but reported a key divergence—sepsis was characterised by pronounced hyperfibrinogenaemia, a thrombogenic clot structure, and marked ex vivo resistance to fibrinolysis, features not typical of ACLF. 102 The same study also found that haemostatic parameters remained remarkably stable over the first 10 days after admission in both groups, suggesting that a single, well-timed measurement may capture the haemostatic state more reliably than the clinical course would suggest. Serial measurement may nevertheless retain value for tracking recovery or deterioration, and the optimal monitoring frequency, the relative informativeness of serial versus single time point sampling, and clinical thresholds for action are not yet defined.

In pretransplant candidates, where PVT prevalence rises with disease severity (Section Portal Vein Thrombosis), TGA—particularly TM-modified ETP and velocity index—has shown preliminary association with thrombotic risk and may eventually contribute to risk-stratified thromboprophylaxis, although prospective validation is required before clinical adoption. 15 Together, these context-specific findings support phenotypic rather than uniform interpretation of TGA in CLD and identify aetiology- and stage-stratified validation as a research priority.

### Fibrin Structure and Fibrinolysis Assays

Fibrin-based assays complement TGA by characterizing the structural and functional consequences of thrombin formation—clot architecture, permeability, and lysis susceptibility—that thrombin kinetics alone cannot capture. The fibrin permeation coefficient (Ks) reflects network porosity and fiber density; lower values indicate compact, lysis-resistant fibrin associated with a prothrombotic phenotype. 89 Turbidimetric assays assess clot formation dynamics, with lag time reflecting protofibril formation and maximum absorbance correlating with fiber thickness and network density. 111 Global fibrinolysis assays such as CLT integrate the effects of fibrin structure, PG, α _2_ -antiplasmin, PAI-1, and TAFI and can identify hyperfibrinolysis or fibrinolytic shutdown even when thrombin generation appears preserved. 16 112

Together, fibrin permeability, turbidity, and fibrinolysis assays provide a structure–function framework that, when integrated with TGA, enables phenotypic stratification—distinguishing preserved thrombin generation with dense, lysis-resistant fibrin from reduced thrombin generation with fragile clots—a paradigm particularly relevant in CLD. 11

Several PG assays exist, each with distinct approaches and limitations. The Nijmegen Haemostasis Assay measures thrombin and plasmin simultaneously in a single well using two noninterfering fluorescent substrates, reporting peak height, fibrin lysis time, and plasmin potential. The simultaneous thrombin and PG (simultaneous thermal analysis) assay uses separate wells with AMC-based fluorometry but lacks a calibrator and does not correct for substrate consumption or inner filter effects. The STPGA (Tarandovsky et al.) 113 measures both analytes in parallel with inner filter correction via the Michaelis–Menten equation, reporting peak plasmin concentration and pre-peak production rate. A common limitation across these methods is incomplete correction for plasmin inhibitors, substrate nonspecificity, and inner filter effects. The calibrated PGA, modeled on calibrated automated thrombography, addresses these shortcomings using an α _2_ -macroglobulin–plasmin complex as calibrator—correcting for substrate exhaustion, inner filter effects, and inhibitor interference—alongside a plasmin-specific substrate (Boc–Glu–Lys–Lys–AMC). It reports lag time, velocity, peak height, time to peak, and endogenous plasmin potential in molar concentrations, with sensitivity to tPA, plasminogen, and α _2_ -antiplasmin, making it the most analytically rigorous method currently available. 80 In a study of 101 patients with cirrhosis (Child–Pugh A/B/C: 36/24/41) and 20 healthy controls, patients demonstrated significantly lower endogenous plasmin potential and plasmin peak, both declining with disease severity, alongside prolonged PG lag time and shorter CLT. Thrombin generation profiles showed no significant differences. TG/PG ratios for endogenous plasmin potential and plasmin peak were elevated in patients and increased progressively with liver disease severity. 114

### Viscoelastic Assays

**Fig. 4:**
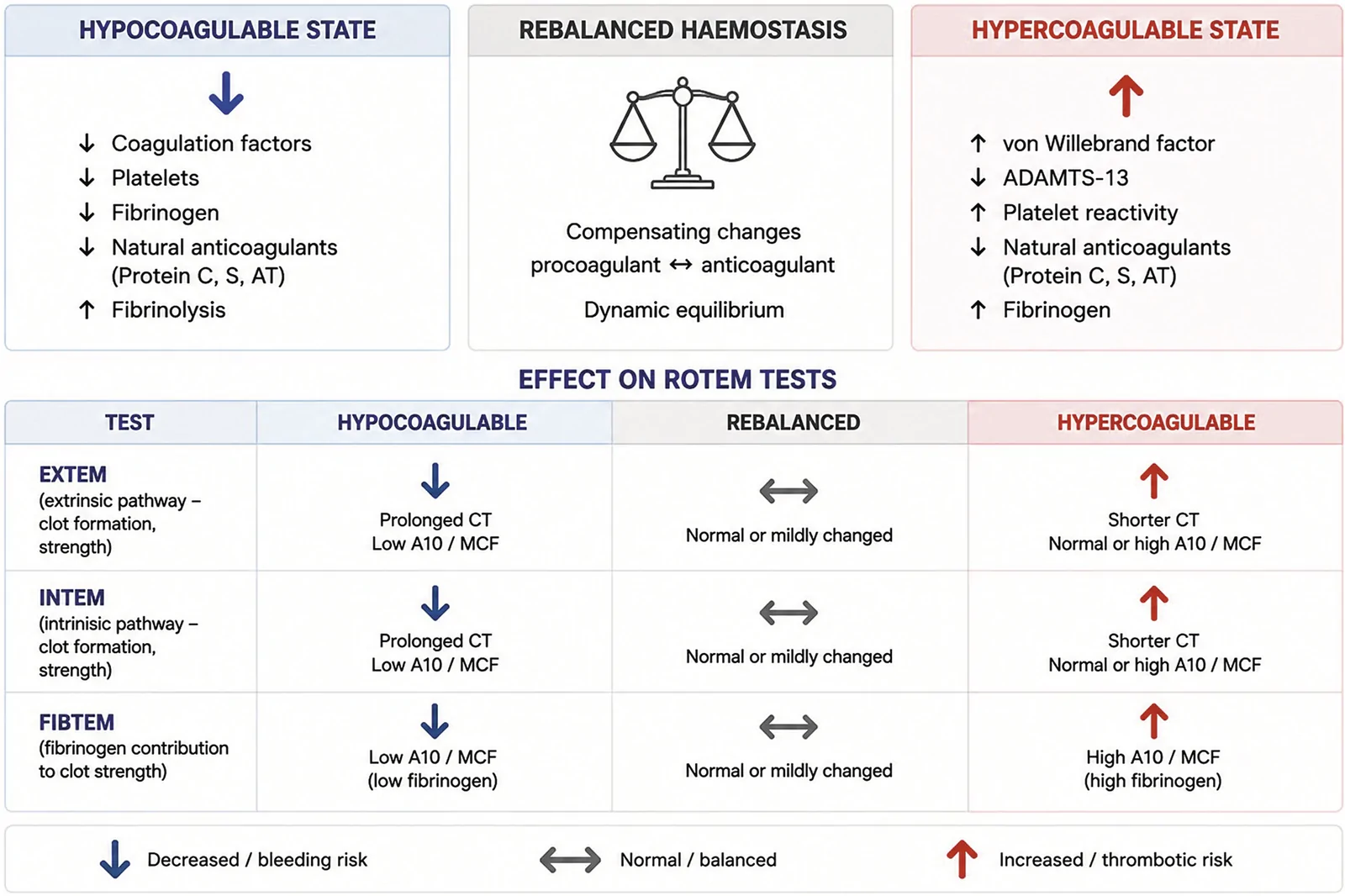
Haemostatic phenotypes and ROTEM findings in cirrhosis. A10, clot amplitude at 10 min; AT, antithrombin; CT, clotting time; EXTEM, extrinsically activated rotational thromboelastometry assay; FIBTEM, fibrinogen-based rotational thromboelastometry assay; INTEM, intrinsically activated rotational thromboelastometry assay; MCF, maximum clot firmness; ROTEM, rotational thromboelastometry.

Viscoelastic assays—thromboelastography (TEG) and rotational thromboelastometry (ROTEM)—provide real-time, whole-blood assessment of clot initiation, propagation, strength, and fibrinolysis, integrating contributions from plasma factors, platelets, and fibrinolytic pathways 110 111 ( Fig. 4 ). Randomised studies have demonstrated that TEG- or ROTEM-guided transfusion strategies reduce blood-product use without increasing bleeding in cirrhotic patients undergoing invasive procedures or presenting with variceal haemorrhage. 115 Comparable findings have been reported in acute liver injury and acute liver failure, where TEG demonstrates minimal net haemostatic impairment despite markedly abnormal conventional tests. 116 These data underpin the strongest existing evidence base for any global assay in CLD transfusion decisions. Two conceptual limitations of viscoelastic testing in CLD nevertheless warrant explicit emphasis. First, viscoelastic assays are largely insensitive to vWF, which compensates for thrombocytopenia by enhancing platelet adhesion under shear; consequently, the platelet contribution measured by TEG/ROTEM tracks platelet count more closely than it does effective in vivo platelet recruitment. Second, these assays do not interrogate the protein C pathway, which is the principal anticoagulant compensation reduced in cirrhosis and the mechanism that drives the rebalanced or hypercoagulable thrombin generation phenotype detected by TM-modified TGA. As a result, viscoelastic testing systematically *underestimates* haemostatic capacity in cirrhosis: a patient with reduced clot amplitude on TEG/ROTEM may nonetheless show preserved or elevated TM-modified ETP, and the discrepancy is greater in compensated disease than in decompensation. 117 Broader implementation is limited by interdevice variability, lack of platform standardisation, and absence of validated cirrhosis-specific thresholds. 91

### Synthesis: Choosing among Global Assays

These assays are complementary rather than interchangeable. TGA provides the most integrated enzymatic readout of procoagulant–anticoagulant balance and is the only widely available test that engages the protein C pathway via thrombomodulin modification—making it uniquely suited to detect the hypercoagulable shift of cirrhosis that conventional tests miss. Fibrin and fibrinolysis assays translate enzymatic potential into clot architecture and lysis susceptibility, identifying patients whose thrombin curves are reassuring but whose clots are structurally fragile or hyperfibrinolytic—a subgroup particularly important in decompensation, infection, and ACLF. Viscoelastic assays are the most clinically actionable at the bedside, integrating platelet and plasma contributions in whole blood with rapid turnaround, and currently carry the strongest evidence for transfusion guidance in cirrhosis. However, they measure clot mechanics rather than enzymatic balance, are insensitive to vWF-mediated platelet recruitment and to the protein C pathway, and therefore systematically underestimate haemostatic capacity in compensated disease. 117 They lack validated cirrhosis-specific thresholds and currently complement rather than replace TGA-based phenotyping.

Despite the conceptual advances offered by TGA, fibrin structural analysis, and viscoelastic testing, no coagulation assay has been validated to independently guide transfusion, prophylaxis, or anticoagulation in cirrhosis. Clinical context remains the primary determinant of management decisions.

## Bleeding in Chronic Liver Disease

### Spontaneous Haemorrhage

Spontaneous haemostasis-related bleeding is defined as an unprovoked haemorrhagic event without an identifiable cause, classified as major or nonmajor according to the ISTH criteria. 118 119 Cirrhosis is associated with increased risk of spontaneous intracranial haemorrhage. 17 Gastrointestinal bleeding—particularly variceal haemorrhage—occurs in up to 30% of cirrhotic patients, with an approximately 15% annual risk in those with large varices. 17 120 Bleeding risk correlates with portal pressure; a hepatic venous pressure gradient (HVPG) >20 mm Hg predicts treatment failure in acute episodes. Variceal prevalence increases with disease severity (42% in Child–Pugh A, 71% in B, 76% in C), and 6-week mortality remains 15–20%. 17 Importantly, portal-hypertensive bleeding is driven primarily by haemodynamic factors rather than intrinsic haemostatic failure, and anticoagulation does not appear to worsen outcomes. 17

### Procedural Bleeding

Periprocedural bleeding risk varies by intervention and is generally low (<1.5–2%) for low-risk procedures, 121 but increases with higher-risk interventions such as endoscopic retrograde cholangiopancreatography, where postprocedure bleeding rates in cirrhosis are 3.6–10.9% versus approximately 1.3% in the general population. 122 123 124 Despite frequent laboratory abnormalities, conventional parameters do not reliably predict bleeding risk. 125 The PROC-BLeeD study identified elevated body mass index and higher MELD scores—not INR—as independent predictors, with bleeding events associated with markedly increased 28-day mortality (hazard ratio [HR]: 6.91; 95% confidence interval [CI]: 4.22–11.31). 126

### Transfusion Strategies and Laboratory ‘Correction’

Observational data from critically ill cirrhotic patients indicate that transfusion-based correction of coagulopathy does not improve outcomes: cryoprecipitate transfusion for very low fibrinogen does not affect bleeding or survival in this population 127 and fresh-frozen plasma (FFP) transfusion during acute variceal haemorrhage is independently associated with increased 42-day mortality, failure to control bleeding, and prolonged hospital stay. 128 The HALT-IT trial showed that tranexamic acid does not reduce mortality in acute gastrointestinal bleeding, including suspected variceal haemorrhage. 129 Consistent with these findings, the upcoming Baveno VIII ongoing consensus discussions are expected to reaffirm that there is no role for routine FFP in its management of variceal bleeding. American Gastroenterological Association guidelines similarly recommend against routine correction of coagulopathy or thrombocytopenia prior to low-risk procedures. 91 130

Transfusion should therefore be individualised, reserving blood-product administration for active bleeding or severe, clinically meaningful deficiencies. Practical thresholds derived from current guidance and randomised evidence include red blood cell transfusion targeting a restrictive haemoglobin threshold of 7 g/dL in stable acute upper gastrointestinal bleeding, 125 platelet transfusion in actively bleeding patients with counts <50 × 10 ^9^ /L (or <30 × 10 ^9^ /L for nonbleeding preprocedural support in selected high-risk procedures), 131 and fibrinogen replacement when levels fall below 1.0–1.5 g/L during active bleeding, 91 121 ideally guided by viscoelastic testing (FIBTEM A5 or A10 on ROTEM, functional fibrinogen on TEG) where available, which provides faster turnaround than Clauss fibrinogen and better reflects whole-blood clot strength. 115 131 Bleeding complications nonetheless remain costly, with the highest incremental hospitalisation costs seen in high-risk surgical settings. 132

## Thrombosis in Chronic Liver Disease

### Venous Thromboembolism

Thrombotic risk in ACLD is increasingly recognised as clinically significant. Early data demonstrated that approximately 0.5% of hospital admissions in cirrhotic patients resulted in a new diagnosis of VTE, comprising 65.5% deep vein thrombosis (DVT), 19.5% pulmonary embolism (PE), and 15% combined events. 133 PVT and systemic VTE are now established as common and clinically relevant complications. PVT occurs in approximately 10 to 25% of patients with cirrhosis, with incidence increasing in parallel with disease severity. 133 134 Among hospitalised cirrhotic patients, VTE prevalence ranges from 0.5% to 1.9%, with reports up to 6.3%. 135 Notably, the risk of VTE is approximately 2.1-fold higher in patients with ACLD compared with those without liver disease. 136 VTE represents an additional complication of liver disease and is also relevant in the post-transplant setting, where vascular events such as hepatic artery thrombosis, PVT, hepatic vein thrombosis, and inferior vena cava thrombosis or stenosis may occur. 17

### Portal Vein Thrombosis

#### Definition and Epidemiology

PVT is defined as partial or complete obstruction of the portal vein or its tributaries and is classified as acute (<6 months) or chronic (>6 months). 17 It is a well-recognised complication of CLD and cirrhosis. 137 PVT is common in cirrhosis, with an overall prevalence of approximately 14% that rises with disease severity. 17 In a cohort of 567 cirrhotic patients, López et al. reported an annual incidence of 3.7%. 137 Reported annual incidence varies widely across cohorts—approximately 3–11% in general cirrhosis populations, rising to 10–17% in decompensated or transplant-candidate populations. 138 The 1-year incidence is markedly higher in Child–Pugh B/C than in Child–Pugh A disease. 139 The prevalence further increases to 26–44% among liver transplant candidates and up to 40% in patients with HCC. 139 140

#### Pathogenesis of Cirrhotic Portal Vein Thrombosis

The pathogenesis of cirrhotic PVT has traditionally been framed through Virchow's triad—reduced portal flow, hypercoagulability, and endothelial injury—with the systemic hypercoagulability of cirrhosis historically taken to be the dominant driver of thrombus formation in the portal circulation. 44 138 Several lines of evidence now challenge this framing and support a reconceptualisation of cirrhotic PVT as predominantly a vascular disease rather than a primarily thrombotic event.

First, established systemic thrombophilias do not behave as risk factors for cirrhotic PVT in the way they do for VTE. Factor V Leiden, the prothrombin G20210A mutation, and non-O ABO blood group—all robust risk factors for DVT—show inconsistent or absent associations with PVT in cirrhotic cohorts. 141 142

The JAK2 V617F mutation, while clearly enriched in noncirrhotic splanchnic vein thrombosis, contributes only modestly in cirrhotic PVT, and routine screening is currently recommended only in noncirrhotic cases. 143 Second, anticoagulation, although it can promote recanalisation and is the basis of current management, is frequently incomplete or ineffective at restoring portal patency in established cirrhotic PVT, even when initiated early and continued at therapeutic intensity. 17 Third, and most strikingly, histopathological examination of portal vein specimens obtained at liver transplantation has shown that a substantial proportion of radiologically defined ‘thrombi’ in cirrhosis consist of organised intimal fibromuscular hyperplasia and chronic vascular remodelling rather than classical fibrin- and platelet-rich thrombus. 21 These findings indicate that what is detected on imaging as PVT may often represent a vasculopathy with luminal remodelling and superimposed flow stasis rather than de novo thrombosis, and they offer a coherent explanation both for the limited efficacy of anticoagulation and for the absence of classical thrombophilic risk factors. 21 144

These observations do not eliminate a haemostatic contribution. Enhanced thrombin generation in the portal circulation has been demonstrated and correlates with disease severity, 77 144 145 gut-derived endotoxaemia upregulates tissue factor expression and may be modulated by nonabsorbable antibiotics such as rifaximin, though outcome data remain limited, 68 146 147 and portal vein endothelial dysfunction—characterised by altered anticoagulant surface properties, inflammatory activation, tissue factor expression, and impaired local fibrinolytic regulation (Section Endothelial Dysfunction)—is increasingly recognised as an active contributor. 21 Rather than overturning the haemostatic component, these findings shift its pathogenic weight: impaired portal flow secondary to portal hypertension and architectural remodelling, together with localised endothelial and intimal abnormalities, now appear to be the dominant drivers in most patients, with systemic hypercoagulability acting as a contributory rather than initiating factor. 21 44 137 138 This reconceptualisation has direct clinical implications, returned to in Section Management, including the recognition that anticoagulation is not uniformly effective for recanalisation in established cirrhotic PVT and that future trials may need to stratify by underlying mechanism—true thrombotic occlusion versus vasculopathic remodelling—rather than by radiological appearance alone.

#### Risk Factors and Prediction

Established risk factors include advanced disease (Child–Pugh B/C), higher MELD score, ascites, and thrombocytopenia, whereas inherited thrombophilia plays a limited role. 17 Several PVT prediction models have been proposed. Liu et al. 148 identified six clinical risk factors (smoking, splenomegaly, esophagogastric varices, prior surgery, red blood cell transfusion, elevated D-dimer) with modest discrimination (area under the curve: 0.70 training, 0.69 validation), whereas Xing et al. developed a nomogram incorporating inflammatory indices with improved performance. 149 More recent models, including Nie et al., still require external validation. 150 These models are limited by small sample sizes, lack of outcome validation, and failure to account for rebalanced haemostasis. Larger prospective multicentre studies are needed. 138

#### Diagnosis

PVT is frequently asymptomatic and detected incidentally on imaging. Doppler ultrasound is firstline, with computed tomography or magnetic resonance imaging used to assess thrombus extent and chronicity, and contrast-enhanced ultrasound to differentiate benign from malignant thrombus. 17

#### Management

Anticoagulation is the cornerstone of PVT management, improving recanalisation, and reducing mortality. Low-molecular-weight heparin (LMWH), vitamin K antagonists (VKAs), and direct oral anticoagulants (DOACs) are all used. DOACs are increasingly accepted in Child–Pugh A, with cautious agent-specific use in Child–Pugh B—where rivaroxaban has historically been avoided, although recent randomised trial data in moderate liver dysfunction may shift this position (see below)—whereas LMWH remains preferred in Child–Pugh C. 17 Anticoagulation does not appear to increase variceal bleeding risk; in the landmark Villa et al. trial, enoxaparin in advanced cirrhosis reduced PVT incidence and delayed hepatic decompensation. 151 The more recent CIRROXABAN trial—the first double-blind, randomised placebo-controlled study of a DOAC in cirrhosis—tested low-dose rivaroxaban in patients with Child–Pugh B7 disease and reported improved portal hypertension complication-free survival without a significant increase in major bleeding, although the trial did not reach its planned sample size and mild bleeding events were more frequent in the rivaroxaban arm. 152 Together, the Villa and CIRROXABAN trials provide the strongest randomised evidence to date that anticoagulation can favorably modify disease course in advanced cirrhosis, although the optimal agent, intensity, and patient-selection criteria remain unresolved. Management is individualised, with interval imaging approximately every 3 months and consideration of endovascular therapy in nonresponders. Transjugular intrahepatic portosystemic shunt is indicated in selected patients with portal-hypertensive complications. Routine prophylactic anticoagulation is not recommended but may be considered in high-risk groups such as transplant candidates or those with prior PVT. 153

## Cardiovascular and Cerebrovascular Risk

ACLD—particularly MASLD with advanced fibrosis—is associated with an increased risk of both ischaemic and haemorrhagic stroke, although the extent to which this represents an independent effect remains uncertain given shared cardiometabolic risk factors. 154 The risk is likely mediated through multiple mechanisms, including cardioembolic pathways, atherosclerosis, microvascular dysfunction, coagulopathy, and systemic inflammation. Consistent with a stiffness-dependent prothrombotic shift, recent thrombin generation data in compensated cirrhosis show that increasing liver stiffness correlates with higher peak thrombin, faster velocity index, and reduced thrombomodulin-mediated ETP inhibition, indicating progressive hypercoagulability even before overt decompensation. 15 Alterations in hepatocyte synthetic function, together with diabetes and visceral adiposity, may further amplify this risk. 44 Less well-established mechanisms have also been proposed, including chronic inflammation (e.g., *Helicobacter pylori* –associated thrombosis) and hepatitis C–related immune complex and cryoglobulin formation. 155 156 157 158 159

Cardiovascular risk in CLD extends beyond stroke. A recent meta-analysis demonstrated a significantly increased risk of myocardial infarction (MI) in patients with MASLD (HR: 1.26, 95% CI: 1.08–1.47), consistent across geographic populations and supporting the need for enhanced cardiovascular surveillance. 160 Population-based data further indicate a transient early increase in *relative* MI risk following cirrhosis diagnosis, although the absolute 1-year risk is not significantly elevated, likely reflecting competing mortality from noncardiovascular causes. 161

Sex-specific differences in cerebrovascular risk have also been reported. In the REGARDS case–cohort study, advanced fibrosis in MASLD was associated with a borderline-significant increase in ischaemic stroke risk in women but not in men (HR: 3.51, 95% CI: 1.00–12.34). 162 Consistent with these findings, a nationwide Taiwanese cohort demonstrated a higher incidence of ischaemic stroke in patients with cirrhosis along with increased poststroke complications and mortality. 163 Meta-analytic evidence further supports an elevated risk of intracranial and subarachnoid haemorrhage in cirrhosis, while associations with ischaemic stroke remain less consistent. 164

Cardiac structural and functional alterations may further contribute to thromboembolic risk. In a study of 184 patients, those with more severe liver disease (MELD ≥ 16) exhibited larger left atrial diameter and higher systolic pulmonary artery pressure; diastolic dysfunction was present in 49.3% of the cohort but did not correlate with MELD score or Child–Pugh class. 165 Consistently, large cohort studies and meta-analyses demonstrate an increased incidence of AF in CLD—particularly in MASLD with advanced fibrosis—with an estimated AF prevalence of 3 to 5% in cirrhosis. 92 93 94 95

## Anticoagulation in Chronic Liver Disease

### Atrial Fibrillation and Stroke Prevention

Patients with cirrhosis and AF are frequently denied anticoagulation due to a presumed bleeding risk, driven in part by overinterpretation of elevated INR values and by bleeding-risk scores such as HAS-BLED, which have not been validated in this population. The European Heart Rhythm Association Practical Guide emphasises that a high HAS-BLED score alone should not preclude anticoagulation; rather, modifiable bleeding-risk factors should be addressed, with closer clinical follow-up in high-risk patients. 45 Current guidance from the ISTH SSC and the 2025 EASL Clinical Practice Guidelines on vascular diseases of the liver supports anticoagulation in Child–Pugh A or B cirrhosis with AF according to standard stroke-prevention recommendations, with DOACs preferred over VKAs. 166 In Child–Pugh C cirrhosis, evidence remains inadequate and most DOACs are contraindicated by product labeling, so decisions should be individualised. 167 Importantly, conventional coagulation parameters (PT/INR, aPTT) do not reliably reflect haemostatic balance in cirrhosis and are not recommended for monitoring anticoagulation, although INR remains a component of Child–Pugh risk stratification. 166 167

Anticoagulation in advanced cirrhosis nevertheless remains challenging due to complex alterations in haemostasis and a limited evidence base. Patients with advanced cirrhosis were excluded from pivotal DOAC trials, and existing data are largely observational. Even so, registry and cohort studies generally suggest that DOACs are associated with reduced stroke without a clear excess of major bleeding compared with no anticoagulation or with VKAs, providing an empirical basis for reconsidering the historical reluctance to anticoagulate. 168 169 Their safety and efficacy in Child–Pugh C cirrhosis, however, remain uncertain and require further investigation. 167

### Antiplatelet Therapy

Evidence regarding antiplatelet therapy is inconsistent, with reports linking its use to both increased bleeding and hepatic decompensation and to reduced mortality and fewer ischaemic events; some studies also suggest a potential association with slower fibrosis progression. These conflicting findings underscore the need for individualised antithrombotic strategies. 170 171 172

### Statins

Statins have emerged as a class with broad pleiotropic effects relevant to CLD, including anti-inflammatory, antifibrotic, and endothelial-modulating actions. 17 Mechanistically, statins enhance hepatic nitric oxide bioavailability, attenuate LSEC dysfunction, and reduce intrahepatic vascular resistance, providing a biological basis for their effects on portal hypertension. Simvastatin has been shown to reduce HVPG in patients with cirrhosis and portal hypertension, 173 and the BLEPS trial demonstrated improved survival—without reduction in rebleeding—when simvastatin was added to standard therapy after variceal bleeding. 174 Safety in advanced disease was established in LIVERHOPE-SAFETY. 175 However, the recent LIVERHOPE-EFFICACY phase 3 trial showed no benefit of simvastatin plus rifaximin over placebo for prevention of ACLF, transplantation, or death in decompensated cirrhosis, 176 suggesting that benefits seen in earlier-stage disease may not extend to advanced decompensation. A specific antithrombotic role remains less well-defined. Observational data suggest reduced PVT incidence in statin users 177 but confounding by indication cannot be excluded and prospective trials are lacking. Larger randomised trials in compensated CLD remain a research priority.

### Towards Individualised Antithrombotic Strategies

The clinical reality of ACLD is that bleeding and thrombotic risks coexist and must be weighed simultaneously rather than sequentially. Current evidence supports several practical principles. First, INR should not be used to predict bleeding or to gate anticoagulation; it is a marker of hepatic synthetic function and a component of severity scores, not a measure of haemostatic balance. 42 91 Second, decisions should integrate disease stage (Child–Pugh, MELD, presence of decompensation, or ACLF), procedure-specific bleeding risk, patient factors (renal function, prior bleeding or thrombosis, frailty), and—where available—global assays. 121 131 Third, where global assays are available, viscoelastic testing currently has the strongest evidence for transfusion decisions, whereas TGA provides mechanistic insight into the pro- and anticoagulant balance but is not yet validated to drive prophylaxis or anticoagulation. 115 Fourth, shared decision-making is essential: in Child–Pugh C disease, AF, or recurrent thrombosis, the tradeoffs are genuinely uncertain, and patient preferences and goals of care should inform management. Until prospective trials validate global assay–guided strategies, the most defensible approach is integrated clinical assessment supported by—but not replaced by—laboratory testing.

## Emerging Mechanisms and Biomarkers

### Hepatokines

Liver-derived signaling proteins increasingly link metabolic regulation to cardiovascular and thrombotic risk. Several hepatokines promote insulin resistance, suggesting that diabetes may mediate part of the association between MASLD and cardiovascular events including stroke. 178 Fetuin-A, fibroblast growth factor 21 (FGF21), and angiopoietin-like protein 8 (ANGPTL8) show promise as biomarkers for early diagnosis and as potential therapeutic targets in MASLD. 179 180

### Fibrosis Severity as a Prothrombotic Driver

Recent data suggest that liver fibrosis severity itself contributes directly to the prothrombotic shift of CLD, independent of decompensation status. In compensated cirrhosis, increasing liver stiffness—measured by transient elastography—correlates with higher peak thrombin, faster velocity index, and reduced thrombomodulin-mediated ETP inhibition on TGA. 15 This suggests that the architectural and biological changes of progressive fibrosis—sinusoidal capillarisation, endothelial activation, microvascular remodelling, and altered haemodynamics—drive a measurable hypercoagulable phenotype before overt clinical decompensation. 181 182 183 If validated in larger prospective cohorts, liver stiffness could become an integrated biomarker linking structural disease severity to coagulation phenotype, with potential utility for identifying patients who might benefit from earlier antithrombotic strategies (e.g., prior PVT prophylaxis in transplant candidates) and for stratifying risk in MASLD-associated cardiovascular disease. 85 151 This represents one of the most promising bridges between hepatology and thrombosis research and is a clear research priority.

## Future Directions and Unresolved Questions

Several priority questions will determine whether global haemostatic assays earn a place in routine clinical practice in CLD.

The dominant obstacle to TGA adoption remains interlaboratory variability. Harmonisation of reagents—tissue factor and phospholipid concentrations, thrombomodulin source and dose—establishment of reference ranges in healthy and cirrhotic populations, definition of preanalytical standards, and validation against bleeding and thrombotic endpoints in adequately powered multicentre cohorts are all needed. Without coordinated international standardisation, even technically robust single-centre studies cannot translate into practice.

Randomised evidence in anticoagulation also remains incomplete. Patients with Child–Pugh C cirrhosis, advanced ACLF, and active decompensation were systematically excluded from pivotal DOAC trials, leaving the highest-risk groups underevidenced. Comparative trials of DOACs versus VKAs and LMWH in Child–Pugh B–C disease, and prospective evaluation of thromboprophylaxis in hospitalised cirrhotic patients beyond the enoxaparin paradigm established by Villa et al., 151 are urgently required. Although viscoelastic-guided transfusion has been tested in randomised trials, equivalent assay-guided trials for TGA-based strategies have not been performed.

Improved PVT prediction is another unmet need. Existing risk models are derived from single-centre cohorts with limited outcome data and do not incorporate rebalanced-haemostasis parameters. External validation, integration of TGA and fibrin-assay readouts, and linkage to portal-haemodynamic markers (HVPG, portal flow velocity) could yield clinically useful tools for transplant-candidate stratification.

MASLD-specific phenotyping deserves particular attention. MASLD is now the leading aetiology of CLD globally and combines metabolic, inflammatory, and prothrombotic drivers—elevated factor VIII, fibrinogen, and PAI-1—that differ qualitatively from viral or alcohol-related disease. 78 110 Systematic coagulation phenotyping across fibrosis stages, with attention to MASH activity and metabolic comorbidity, remains a major gap. Liver stiffness may emerge as an integrated marker of haemostatic phenotype. Recent evidence that liver stiffness correlates with TGA parameters in compensated disease 15 suggests that transient elastography may serve as a noninvasive surrogate for haemostatic state. Prospective validation linking stiffness measurements to clinical thrombotic events would have immediate translational value.

Circulating hepatokines, including fetuin-A, FGF21, and ANGPTL8, similarly require prospective validation as predictors of thrombotic and cardiovascular events in CLD before clinical adoption can be considered.

Preanalytical and cellular-component standardisation is a further methodological priority. Whole-blood TGA, viscoelastic testing, and fibrin-based assays are all sensitive to anticoagulant choice, time to processing, temperature, and platelet count. Standardised protocols specific to CLD populations are needed, particularly in patients with anaemia and severe thrombocytopenia where conventional cut-offs may not apply.

Finally, endothelial and vascular biology remains undercharacterised. Portal vein endothelial dysfunction is mechanistically attractive but difficult to study owing to anatomical inaccessibility. 21 77 Translational approaches using transplant explants, organoid systems, and circulating endothelial biomarkers may close this gap.

Addressing these questions will require sustained collaboration between haematologists, hepatologists, intensivists, and transplantation specialists, supported by international assay-standardisation programmes and adequately funded prospective trials.

## Conclusion

CLD haemostasis is not hypocoagulable but rebalanced, fragile, and bidirectionally unstable. Conventional coagulation tests assess only a fraction of in vivo haemostasis and frequently misrepresent both bleeding and thrombotic risk. Global assays—TGA, fibrin and fibrinolysis testing, and viscoelastic methods—provide complementary mechanistic and functional information and have reshaped the conceptual understanding of CLD coagulopathy. None, however, has yet been validated to independently guide transfusion, prophylaxis, or anticoagulation decisions in routine practice.

Both bleeding and thrombotic complications carry meaningful clinical weight. Bleeding in cirrhosis is most often a consequence of portal hypertension rather than haemostatic failure; thrombosis—particularly PVT, VTE, and AF-related stroke—is increasingly recognised and frequently undertreated. Management must therefore be individualised, integrating disease stage, procedure-specific risk, patient factors, and shared decision-making, with conventional and global assays informing rather than dictating clinical choices.

The most promising frontiers—TGA standardisation and clinical validation, MASLD-specific phenotyping, liver stiffness as an integrated biomarker, and prospective anticoagulation trials in advanced disease—sit squarely at the interface of haematology and hepatology. Closing these gaps will demand coordinated international effort. The clinical reward is a shift from one-size-fits-all coagulation correction to genuinely individualised, mechanism-informed thrombosis and bleeding care for patients with CLD.

## Data Availability

Data sharing is not applicable to this article, as no new data were created or analysed.
